# Supplementary material for: From sequence to enzyme mechanism using multi-label machine learning
Source: BMC Bioinformatics. 2014 May 19;15:150. doi: 10.1186/1471-2105-15-150 (PMC4229970; doi:10.1186/1471-2105-15-150)
Supplement: Additional file 2 — Java code of ml2db. Additional file ml2db_code.tar.gz contains the Java source code to run the multi-label machine learning experiments and save the results to database. The code’s Javadoc is included. [file 1471-2105-15-150-S2.zip › additional file 2/ml2db/ml2db/doc/mulan/data/package-frame.html]

mulan.data


# mulan.data

## Interfaces

- *LabelClustering*
- *LabelNode*
- *LabelPairsDependenceIdentifier*
- *LabelsMetaData*
- *Stratification*

## Classes

- ConditionalDependenceIdentifier
- ConverterCLUS
- ConverterLibSVM
- DataUtils
- GreedyLabelClustering
- IterativeStratification
- LabelNodeImpl
- LabelPowersetStratification
- LabelsBuilder
- LabelSet
- LabelsMetaDataImpl
- LabelsPair
- MultiLabelInstances
- Statistics
- UnconditionalChiSquareIdentifier

## Exceptions

- DataLoadException
- InvalidDataFormatException
- LabelsBuilderException
